# Modified blink dynamic index predicts activity and severity in patient with facial nerve palsy

**DOI:** 10.3389/fopht.2022.960593

**Published:** 2022-08-19

**Authors:** Yuri Kim, Helen Lew

**Affiliations:** Department of Ophthalmology, CHA Bundang Medical Center, Seongnam, South Korea

**Keywords:** blink pattern, blink dynamics, facial nerve palsy, modified IPF, modified CS

## Abstract

**Purpose:**

We analyzed the dynamics of blink and defined the blink index in facial nerve palsy (FNP) patients using an ocular surface interferometer associated with clinical characteristics and clinical progress.

**Methods:**

In total, 49 patients were enrolled this study. All patients were tested using an ocular surface interferometer which is used to measure blink patterns (total blink (TB), partial blink rate (PBR)) and blink dynamics (blink time (BT), lid closing time (LCT), closure time (CT), lid opening time (LOT), interblink time (IBT), closing speed (CS), and opening speed (OS)) using 600 frames recorded over 20 s. The distance of lagophthalmos and modified interpalpebral fissure (IPF), which was defined as the actual moving distance of the upper eyelid, subtracting the distance of lagophthalmos from IPF, was measured using the ImageJ program. The types of FNP were “idiopathic” (Bell’s palsy) and “surgical” (following the neurosurgery). Patients were classified into “acute” and “chronic” based on the duration of 6 months from the onset time of FNP. The clinical characteristics were classified into mild or severe according to the severe degree of exposure keratopathy—as “severe” if treatment such as tarsorrhaphy and gold plate insertion was required.

**Results:**

Reduced MRD_1_, brow height, and modified IPF and increased lagophthalmos were noted in the palsy side. LCT was longer and CS, modified CS, and modified OS were shorter in the palsy side. The LCT was longer and the modified CS was shorter in idiopathic patients with acute onset and with clinically severe. IBT was increased in idiopathic patients with clinically severe.

**Conclusion:**

Analyzing the blink patterns and blink dynamics, this study suggests meaningful indicators among blink profiles and dynamics, LCT, and modified CS based on modified IPF. It was more associated in the idiopathic type than in the surgical type of FNP patients. The modified CS can be a useful tool for evaluating the activity and severity indicator of FNP.

## Introduction

Facial nerve palsy (FNP) can occur as a result of various medical conditions such as idiopathic, infectious, traumatic, and neoplastic (1, 2). The most common disease causing FNP is known as idiopathic Bell’s palsy (1). It affects 20–30 persons per 100,000 annually, and one in 60 individuals will be affected over the course of their lifetime (3, 4).

5 reported that normal blink consists of relaxation of the levator palpebrae muscle and contraction of the orbicularis oculi muscle. The orbicularis oculi muscle, which mainly works for blink, is innervated by the facial nerve. FNP can cause loss of facial expressive motor function, which presents upper and lower face weakness including the orbicularis muscle, causing related features including distorted face, blink impairment, affecting communication, and esthetics (6)

Regarding eye manifestations, FNP includes the inability to elevate the eyebrow causing a droopy eyelid, incomplete eye closure leading to exposure keratopathy, tearing or reflex tearing due to lacrimal pump failure, and eyelid retraction combined with midface ptosis or facial asymmetry presenting ectropion with lid laxity for aging (7). Mostly, patients suffer from blurred vision and ocular discomfort due to deficits in blink and eye closure (8). Therefore, clinicians should diagnose it and take action for the eye protection for FNP patients with impaired eye closure (9).

However, most patients with FNP are usually referred to a neurology or otolaryngology doctor. Therefore, proper management of ophthalmic damage could be delayed for this reason. For patients who visit the ophthalmologist even belatedly, an objective, precise, and repetitive diagnostic tool for the evaluation of blink reflex is needed. Hence, we hereby tried to investigate blink reflex, in order to evaluate for early detection and clinical prognosis by conducting a video analysis programmed by ocular surface interferometry, which can be easily performed in the outpatient clinic.

It is well known that paralytic symptoms could relieve over time, but the characteristics of eyelid blink and clinical progress are yet to be studied thoroughly. Thus, we aimed to analyze the dynamics of blink and define the novel blink index, in order to evaluate the correlation of clinical characteristics and clinical course in FNP patients using an ocular surface interferometer.

## Materials and methods

### Patients and clinical evaluations

This study and data collection protocol were approved by the Institutional Review Board of CHA Bundang Medical Center (CHAMC IRB 2018-12-016-002), and this study adhered to all relevant tenets of the Declaration of Helsinki. We retrospectively reviewed the medical chart and analyzed 52 FNP patients who visited our ophthalmic clinic from April 2017 to March 2022. This retrospective study used data from the medical records of etiology of FNP including iatrogenic, trauma, tumor, surgery, and infection including Ramsey–Hunt syndrome. Patients with blepharospasm, secondary dystonia, dry eye syndrome, and other conditions of ocular disease, which can cause abnormal blink of the periocular muscles, were excluded.

The types of FNP were classified into “idiopathic” (Bell’s palsy) and “surgical” (following the neurosurgery). Patients were classified into “acute” and “chronic” based on the duration of 6 months from the onset time of FNP. The clinical characteristics were classified into mild or severe according to the severe degree of exposure keratopathy—as “severe” if treatment such as tarsorrhaphy and gold plate insertion was required.

Periocular topographic measurement included MRD_1_ (upper margin reflex distance), MRD_2_ (lower margin reflex distance), and Brow height measured by a single examiner (H Lew). The distance of lagophthalmos, IPF, and modified IPF was measured using the ImageJ program ver. 1.53 (National Institutes of Health) by a single examiner (Y Kim). The mean corneal diameter (11.6 mm) was used as a reference to set the scale for each photograph. Here, the modified IPF was defined as the actual moving distance of the upper eyelid, excluding the distance of lagophthalmos from IPF. All subjects could be tested using a LipiView^®^ Interferometer (TearScience, Morrisville, NC, USA). The control group was defined as the opposite eye of each patient.

### Blink dynamics analysis

An analysis of blink dynamics was based on the 20 s of videos (600 frames), which were recorded using the ocular surface interferometer. The total blink (TB,/20s) and partial blink ratio (PBR, %) was counted using the internal program of the ocular surface interferometer.

We set the blink cycle as the blink time (BT), which means the time during the upper eyelid closing movement, and interblink time (IBT), which means keeping the upper eyelid opened. BT was defined as the summation of lid closing time (LCT) taken by the interpalpebral fissure (IPF) to reach the maximum closure from the minimum closure, lid opening time (LOT) as the time taken by the upper eyelid to change from the minimum to maximum IPF, and closure time (CT) as the time for which the upper eyelid remained completely closed.

**Figure 1:**
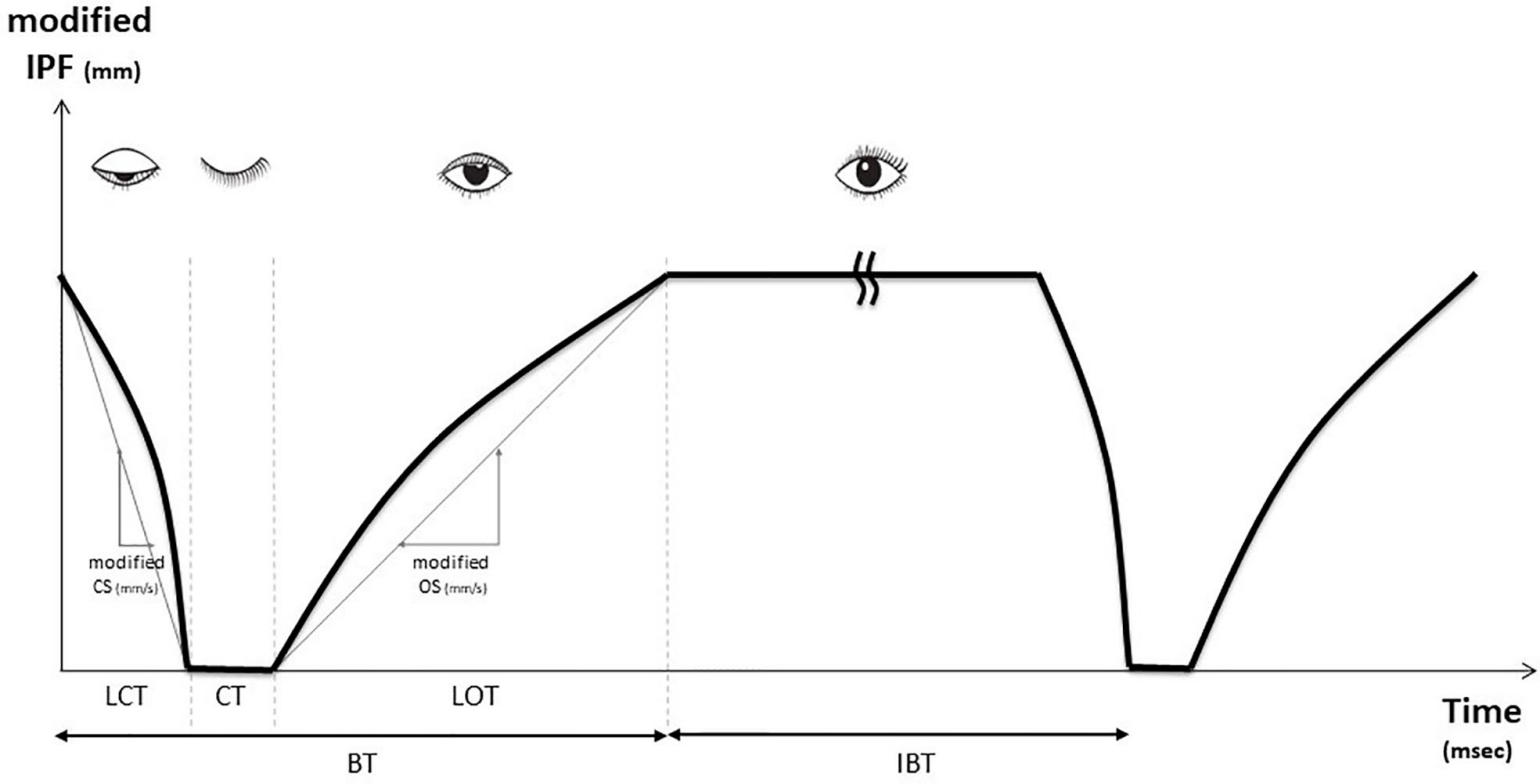
Blink profiles obtained using ocular surface interferometer (LipiView^®^) Blink time (BT) was defined as summation of lid closing time (LCT) taken by the interpalpebral fissure (IPF) to reach the maximum closure from the minimum closure, lid opening time (LOT) as the time taken by the upper eyelid to change from the minimum to maximum IPF, and closure time (CT) as the time for which the upper eyelid remained completely closed. We set the blink cycle as the summation of blink time (BT) and the interblink time (IBT) which means the time of keeping the eyelid open. A kink is inserted during IBT. During the LCT period of the LOT period, at least two time points were calculated to estimate the curvature of the blink dynamics curve. Closing speed (CS) and opening speed (OS) were calculated as IPF per LCT (mm/s) and IPF per LOT (mm/s) and modified CS and modified OS were calculated as modified IPF per LCT (mm/s) and modified IPF per LOT (mm/s).

A blink dynamic curve could be drawn from these data. During both the period of LCT and LOT, at least two time points were picked and calculated to estimate the curvature of the blink dynamic curve. The blink dynamic index of closing and opening eyelid was defined as closing speed (CS), opening speed (OS), modified closing speed (modified CS), and modified opening speed (modified OS). CS and OS were calculated as IPF per LCT (mm/s) and IPF per LOT (mm/s), while modified CS and modified OS were calculated as modified IPF per LCT (mm/s) and modified IPF per LOT (mm/s). All measurements were performed on all blinks made within the 20 s and averaged. Each blink curve was drawn based on the modified IPF versus time graph (**Figure 1**). A desktop computer running Windows 10 and video software was used for data capture and analysis. All measurements were derived by a single examiner (Y Kim).

### Statistical analysis

All statistical analyses employed SPSS for Windows, version 27.0 (IBM Corp., Armonk, NY, USA). Paired t-test and Mann–Whitney test were used to compare the palsy side and control, acute onset patient and chronic onset patient, and clinically mild and severe patient. A p value <0.05 was regarded as statistically significant. We reported dichotomous outcomes as odds ratios (ORs) and continuous outcomes as the mean and their respective 95% confidence intervals (CIs) with the chi-square test. Binary logistic regression analysis was used to find the relationship between the dependent variables.

## Results

A total of 49 FNP patients (21 of men, 27 of women) who met the inclusion and exclusion criteria were enrolled in this study. The mean age of onset was 48.45 ± 17.70 years. The average onset of FNP was 49.92 ± 80.25 months from the first visit of the ophthalmology clinic. Thirty-nine patients were diagnosed as idiopathic such as Bell’s palsy, 13 as surgical FNP such as postoperatively (orbitozygomaticomaxillary fracture operation, Burr hole operation due to brain hemorrhage, temporal bone fracture operation) (**Table 1**).

**Table 1:** Demographics of FNP patients.

	Total (n=49)
Sex (M:F)	21 : 27
Onset age (years)	48.45 ± 17.70
Site (OD : OS)	27 : 22
Onset (mon)	96.20 ± 161.79
Causes (n, %)	
Idiopathic	36 (73)
Surgical	13 (27)

According to eyelid topographics, MRD_1_ and brow height were significantly reduced in the palsy side compared to the normal side in each patient. The distance of lagophthalmos was longer and modified IPF was shorter in the palsy side (**Table 2**).

**Table 2:** Eyelid topographics of the FNP patients.

	Palsy side (n=49)	Normal side (n=49)	Total (n=49)	*p value*
MRD_1_ (mm)	2.29 ± 1.53	3.41 ± 1.05	2.85 ± 1.42	***0.003***
MRD_2_ (mm)	4.36 ± 1.04	4.09 ± 0.73	4.22 ± 0.90	*0.235*
Brow (mm)	0.57 ± 0.64	1.91 ± 2.11	1.24 ± 1.99	***0.007***
IPF (mm)	6.64 ± 1.94	7.50 ± 1.47	1.24 ± 1.99	*0.063*
Lago (mm)	4.21 ± 3.29	2.82 ± 3.00	2.36 ± 3.11	***<0.001***
Modified IPF (mm)	2.82 ± 3.00	5.25 ± 4.39	4.03 ± 3.94	***<0.001***

IPF, interpalpebral fissure. Bold: p < 0.05.

Regarding blink pattern, the PBR was about two times higher in the palsy side. When measuring blink dynamics, most of the blink dynamics data had a statistically significant difference. BT, CT, LOT, CS, and modified CS and modified OS were shorter, and LCT was longer. There seems to be no difference in CS, but the modified CS decreased significantly in the palsy side (**Table 3**).

**Table 3:** Blink profiles of the FNP patients .

	Palsy side (n=49)	Normal side (n=49)	Total (n=49)	*p value*
***Blink pattern***					
TB (/20s)	7.34 ± 4.37	8.23 ± 4.71	7.78 ± 4.55	*0.205*	
PBR (%)	80.87 ± 27.58	46.40 ± 33.80	63.63 ± 35.28	***<0.001***	
***Blink dynamics***					
BT (ms)	740.7 ± 221.9	816.5 ± 186.0	778.6 ± 207.7	***0.001***	
LCT (ms)	232.0 ± 123.0	174.1 ± 50.9	203.0 ± 98.2	***<0.001***	
CT (ms)	85.0 ± 59.2	114.6 ± 80.3	99.8 ± 71.9	***<0.001***	
LOT (ms)	423.6 ± 144.8	527.8 ± 151.1	475.7 ± 156.5	***<0.001***	
IBT (ms)	9223.4 ± 3400.4	9223.4 ± 4462.7	9223.4 ± 4011.3	*0.082*	
CS (mm/s)	40.76 ± 20.29	49.16 ± 21.38	45.0 ± 21.2	*0.084*	
OS (mm/s)	52.63 ± 138.76	18.37 ± 6.99	35.5 ± 99.5	***<0.001***	
Modified CS (mm/s)	21.89 ± 16.82	45.97 ± 20.65	33.9 ± 22.3	***<0.001***	
Modified OS (mm/s)	19.02 ± 26.91	17.41 ± 7.45	18.2 ± 19.7	***<0.001***	

TB, total blink; PBR, partial blink ratio; BT, blink time; LCT, lid closing time; CT, closure time; LOT, lid opening time; IBT, interblink time; CS, closing speed; OS, opening speed. Bold: p < 0.05.

Based on the onset and the cause of FNP, the ratio compared to the normal side, MRD_2_, IPF, and LCT were longer, and the brow height and modified CS were shorter in acute patients. The more chronic the process becomes, the shorter MRD_2_ and the longer the brow height and the modified IPF in idiopathic and total patients. However, it is opposite in surgical patients, whose MRD_2_ tends to become longer and whose brow height and modified IPF tend to be shorter. No definite difference was found in blink pattern. The onset (acute and chronic) and the cause (idiopathic and surgical) had a correlation (odds ratio [OR] = 0.40; 95% confidence interval [CI] = 0.21–0.76) (p = 0.004). It was found that LCT recovered shortly and modified CS recovered to 36% in the acute patients compared with the chronic patients at 69% according to the normal side in idiopathic patients. However, it did not turn out the same in surgical patients. The more chronic the progress becomes, the longer the LCT and less modified CS recover compared to the normal side (**Table 4**).

**Table 4:** Eyelid topographic measurement and blink profiles according to the onset of FNP and causes.

Ratio	Idiopathic (n=36)			Surgical (n=13)			Total (n=49)		
	Acute (n=25)	Chronic (n=11)	*p value*	Acute (n=9)	Chronic (n=4)	*p value*	Acute (n=20)	Chronic (n=29)	*p value*
Onset age (years)	52.92 ± 16.56	45.76 ± 21.80	*0.491*	48.00 ± 7.12	49.50 ± 9.71	*0.816*	51.60 ± 13.15	46.28 ± 20.48	*0.839*
MRD_1_	1.05 ± 1.03	0.57 ± 0.37	*0.152*	0.52 ± 0.38	0.75 ± 0.69	*0.648*	0.86 ± 0.89	0.62 ± 0.44	*0.451*
MRD_2_	1.10 ± 0.13	0.91 ± 0.14	***0.008***	1.11 ± 0.13	1.33 ± 0.80	*0.638*	1.11 ± 0.12	1.03 ± 0.43	***0.009***
Brow	-0.20 ± 0.45	0.47 ± 0.46	***0.045***	0.21 ± 0.42	-0.22 ± 0.69	*0.558*	-0.02 ± 0.46	0.21 ± 0.62	*0.215*
IPF	1.05 ± 0.21	1.00 ± 0.21	***0.049***	0.95 ± 0.11	1.12 ± 0.20	***0.018***	1.01 ± 0.17	1.03 ± 0.21	*0.986*
Lago	2.54 ± 2.55	2.87 ± 2.09	*0.83*	3.46 ± 3.38	2.14 ± 0.00	*1*	3.00 ± 2.50	2.81 ± 1.99	*0.896*
Modified IPF	0.58 ± 0.36	0.75 ± 0.51	*0.214*	0.29 ± 0.25	0.62 ± 0.36	***0.041***	0.45 ± 0.35	0.72 ± 0.48	***0.011***
***Blink pattern***									
TB	0.88 ± 0.44	1.22 ± 0.91	*0.287*	0.87 ± 0.39	1.53 ± 1.88	*0.597*	0.88 ± 0.42	1.31 ± 1.25	*0.183*
PBR	1.54 ± 0.71	2.66 ± 3.95	*0.773*	2.01 ± 0.95	2.99 ± 2.93	*0.837*	1.79 ± 0.86	2.75 ± 3.68	*0.598*
***Blink dynamics***									
BT	1.0 ± 0.3	1.0 ± 0.3	*0.613*	0.8 ± 0.2	0.9 ± 0.3	*0.187*	0.9 ± 0.3	1.0 ± 0.3	*0.515*
LCT	1.6 ± 0.8	1.2 ± 0.6	***0.006***	1.3 ± 0.6	1.7 ± 0.5	***0.005***	1.5 ± 0.8	1.3 ± 0.6	*0.295*
CT	1.1 ± 0.8	0.9 ± 0.4	*0.502*	0.7 ± 0.3	0.7 ± 0.6	*0.761*	0.9 ± 0.6	0.9 ± 0.5	*0.303*
LOT	0.8 ± 0.3	1.0 ± 0.4	*0.219*	0.7 ± 0.2	0.8 ± 0.4	*0.97*	0.8 ± 0.3	0.9 ± 0.4	*0.171*
IBT	1.8 ± 1.8	1.2 ± 0.8	*0.402*	1.2 ± 0.3	1.2 ± 0.4	*0.734*	1.5 ± 1.4	1.2 ± 0.7	*0.395*
CS	0.8 ± 0.3	1.0 ± 0.4	*0.087*	0.9 ± 0.4	0.8 ± 0.3	*0.678*	0.8 ± 0.3	0.9 ± 0.4	*0.152*
OS	1.8 ± 1.6	2.7 ± 4.5	*0.229*	3.6 ± 9.1	2.7 ± 2.9	*0.327*	2.7 ± 6.5	2.7 ± 4.1	*0.477*
Modified CS	0.4 ± 0.3	0.7 ± 0.6	***0.049***	0.4 ± 0.4	0.3 ± 0.2	*0.94*	0.4 ± 0.3	0.6 ± 0.5	*0.054*
Modified OS	1.0 ± 1.2	1.9 ± 3.7	*0.542*	0.8 ± 0.7	0.9 ± 0.5	*0.598*	0.9 ± 1.0	1.6 ± 3.2	*0.286*

TB, total blink; PBR, partial blink ratio; BT, blink time; LCT, lid closing time; CT, closure time; LOT, lid opening time; IBT, interblink time; IPF, interpalpebral fissure; CS, closing speed; OS, opening speed. Bold: p < 0.05 (odds ratio [OR] = 0.40; 95% confidence interval [CI] = 0.21 0.76) (p = 0.004).

Based on the severity and cause of FNP, the ratio compared to the normal side, MRD_2_, and IPF were longer in severe patients. With regard to blink pattern, TB was lower in severe patients. These patterns were similar in idiopathic and surgical patients. In blink dynamics, longer LCT and IBT and a shorter modified CS were distinct in severe patients. LOT was shorter and OS was higher in severe patients, which is statistically significantly different in idiopathic patients. However, the data of surgical patients were different with idiopathic patients, so in the total patients’ data, regarding the severity of FNP, it did not turn out to be statistically significantly different. Regarding the severity of FNP, mild patients had about 72% and severe patients had about 36% more slowly modified CS than normal patients. In idiopathic FNP patients, there were some data with a statistically significant difference. However, there was no prominent difference in surgical patients (**Table 5**).

**Table 5:** Eyelid topographic measurement and blink profiles according to the severity of FNP and causes.

Ratio	Idiopathic (n=36)			Surgical (n=13)			Total (n=49)		
	Mild (n=25)	Severe (n=11)	*p value*	Mild (n=4)	Severe (n=9)	*p value*	Mild (n=34)	Severe (n=15)	*p value*
Onset age (years)	46.84 ± 21.50	52.09 ± 18.32	*0.47*	51.25 ± 8.77	47.22 ± 7.24	*0.486*	47.45 ± 20.17	49.90 ± 14.31	*0.959*
MRD_1_	0.97 ± 0.98	0.57 ± 0.35	*0.43*	0.55 ± 0.07	0.60 ± 0.52	*1*	0.92 ± 0.92	0.59 ± 0.45	*0.333*
MRD_2_	0.97 ± 0.15	1.16 ± 0.15	***0.048***	1.04 ± 0.41	1.21 ± 0.43	*0.788*	0.98 ± 0.18	1.19 ± 0.34	***0.034***
Brow	0.33 ± 0.44	-0.33 ± 0.58	*0.095*	0.17 ± 0.24	-0.03 ± 0.65	*0.522*	0.30 ± 0.40	-0.15 ± 0.60	*0.084*
IPF	0.95 ± 0.17	1.12 ± 0.23	***0.007***	1.02 ± 0.13	1.06 ± 0.22	*0.748*	0.97 ± 0.16	1.10 ± 0.23	***0.013***
Lago	3.10 ± 2.44	2.53 ± 1.75	*0.631*	1.08 ± 0.00	3.99 ± 2.63	*0.221*	2.81 ± 2.35	2.90 ± 1.91	*1*
Modified IPF	0.70 ± 0.32	0.63 ± 0.67	*0.086*	0.44 ± 0.39	0.43 ± 0.32	*0.964*	0.64 ± 0.35	0.53 ± 0.53	*0.09*
***Blink pattern***									
TB	1.32 ± 0.92	0.77 ± 0.40	***0.018***	1.46 ± 1.85	0.91 ± 0.36	*0.762*	1.37 ± 1.27	0.83 ± 0.38	***0.024***
PBR	1.72 ± 1.93	3.35 ± 4.79	*0.148*	2.74 ± 2.93	2.26 ± 1.15	*0.441*	2.03 ± 2.30	2.91 ± 3.76	*0.085*
***Blink dynamics***									
BT	1.0 ± 0.4	0.9 ± 0.3	*0.584*	0.9 ± 0.2	0.9 ± 0.2	*0.791*	1.0 ± 0.3	0.9 ± 0.3	*0.565*
LCT	1.2 ± 0.7	1.6 ± 0.8	***0.008***	1.6 ± 0.6	1.4 ± 0.6	*0.307*	1.3 ± 0.7	1.5 ± 0.7	***0.049***
CT	1.0 ± 0.6	1.0 ± 0.5	*0.561*	0.8 ± 0.6	0.6 ± 0.3	*0.351*	0.9 ± 0.6	0.8 ± 0.4	*0.641*
LOT	1.0 ± 0.4	0.8 ± 0.3	***0.016***	0.7 ± 0.3	0.8 ± 0.3	*0.265*	0.9 ± 0.4	0.8 ± 0.3	*0.12*
IBT	1.2 ± 1.3	1.6 ± 1.1	***0.035***	1.1 ± 0.3	1.2 ± 0.3	*0.835*	1.2 ± 1.1	1.4 ± 0.9	***0.038***
CS	1.0 ± 0.5	0.9 ± 0.3	*0.462*	0.8 ± 0.2	0.9 ± 0.4	*0.925*	0.9 ± 0.4	0.9 ± 0.3	*0.612*
OS	2.4 ± 4.4	2.5 ± 2.9	***0.033***	4.6 ± 9.0	1.5 ± 0.8	*0.157*	3.1 ± 6.2	2.1 ± 2.3	*0.191*
Modified CS	0.7 ± 0.5	0.4 ± 0.5	***0.002***	0.4 ± 0.2	0.4 ± 0.3	*0.692*	0.6 ± 0.5	0.4 ± 0.4	***0.004***
Modified OS	1.9 ± 3.9	1.1 ± 1.3	*0.338*	1.0 ± 0.8	0.6 ± 0.4	*0.146*	1.6 ± 3.3	0.9 ± 1.0	*0.097*

TB, total blink; PBR, partial blink ratio; BT, blink time; LCT, lid closing time; CT, closure time; LOT, lid opening time; IBT, interblink time; IPF, interpalpebral fissure; CS, closing speed; OS, opening speed. Bold: p < 0.05.

**Figure 2:**
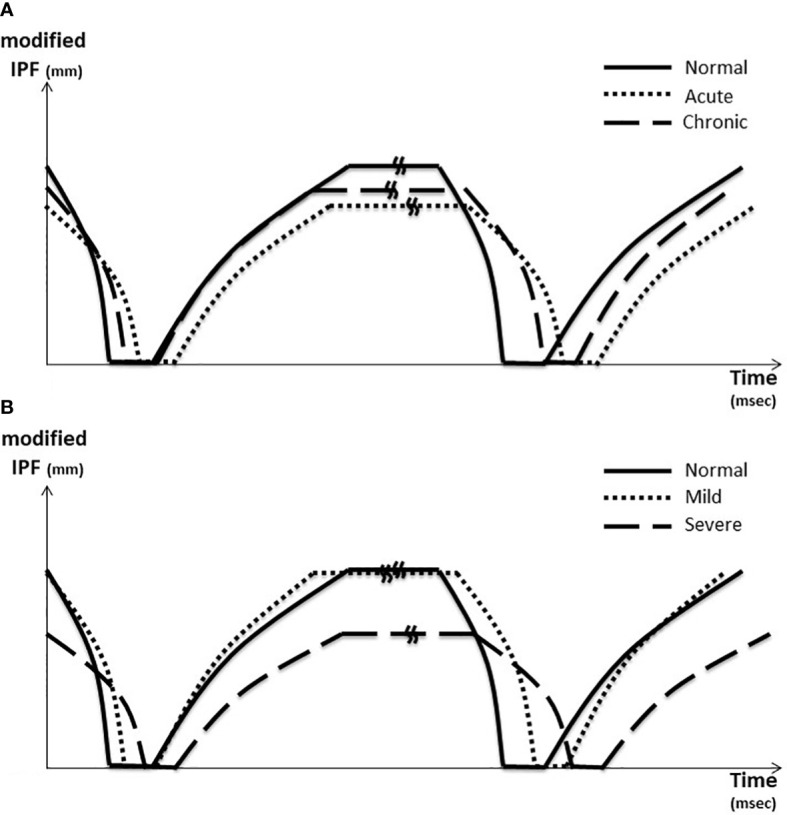
Blink dynamics graph. **(A)** According to the onset of FNP. **(B)** According to the severity of FNP In **(A)**, shorter modified IPF and decreased modified CS and modified OS were noted in acute patient (dotted line). The tangent of each graph, defined as modified CS and OS, was reduced in the acute patient (dotted line) compared to the normal patient (solid line). The tangent recovered to normal in the chronic patient (long dotted line). In **(B)**, shorter modified IPF and decreased modified CS and modified OS were noted in the severe patient (long dotted line). The tangent of each graph, defined as modified CS and OS, was reduced in the severe patient (long dotted line) compared to the normal patient (solid line). The tangent recovered to normal in the mild patient (dotted line). A kink is inserted in the middle of the IBT line, which was omitted in consideration of each ratio. IPF, interpalpebral fissure; CS, closing speed; OS, opening speed.

**Figure 3:**
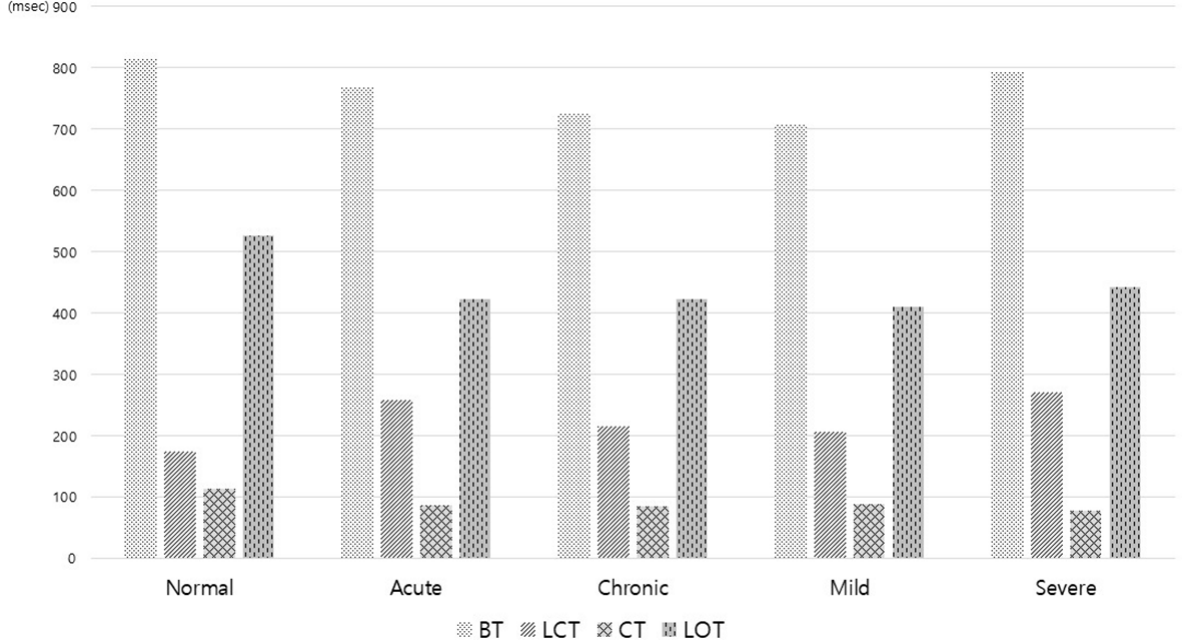
Three components of blink time consisting of lid closing time (LCT), closure time (CT), and lid opening time (LOT) in the facial nerve palsy patients according to activity and severity. BT of the FNP side decreased to 740.7 ms from 816.5 ms of the normal side. BT from the palsy side included significantly elongated LCT about 33% (174.1 to 232.0 ms) and shortened CT about 35% and LOT about 25%. This finding was prominent in acute and severe FNP patients.

Shorter modified IPF and decreased modified CS and modified OS, as the tangent of each graph, were found in acute patients and severe patients. Longer modified IPF and increased modified CS and modified OS were noted in the normal group (**Figure 2**). As for the three components of BT, consisting of LCT, CT, and LOT, BT of the FNP side decreased to 740.7 ms from 816.5 ms of the normal side, which included significantly elongated LCT about 33% (174.1 to 232.0 ms) and shortened CT about 35% and LOT about 25%. This finding was prominent in acute and severe FNP patients (**Figure 3**).

## Discussion

This study was aimed to identify the dynamic blink profiles of FNP. Even though there have been many methods to assess the eyelid function of FNP, there are few tools related to blink movement. Recently, some studies have been performed to use video frame analysis to assess blink dynamics and blink pattern which can be applied in the outpatient clinic. According to the latest findings, the EMG signals of the facial muscle or fraction amplitude of low-frequency fluctuation (fALFF) using high-density surface electromyography have been used recently (10, 11).

Through the authors’ blink patterns and blink dynamics analysis, patients with FNP presented an increased number of incomplete blinks. The modified OS and modified CS using modified IPF among blink indices were derived as meaningful indicators. The difference in IPF between the palsy side and normal side was about 0.86 mm, but the difference in modified IPF was about 2.43 mm compared with the normal side (**Table 2**).

In general, there seems to be no difference in CS and OS, but the modified CS and modified OS were statistically significantly different. The tangent of each graph, defined as modified CS and OS, was reduced in the palsy side compared to the normal side. The tangent recovered to normal in the mild and chronic patients. It indicated that modified CS had a strong correlation with the severity of the disease and the clinical activity of the idiopathic FNP (**Figure 2**). In this study, modified CS was calculated about half to one-third as high as the modified OS, which was similar to the previous results of 5 reported by orbicularis oculi electromyography (OO-EMG) (5, 12). The patients with a more acute onset and more severe corneal damage turned out to have a slower modified CS. The acute-onset patients could be clinically severe, while the chronic-onset patients could be clinically mild. It was found that there was a correlation between onset and clinical progress ([OR] = 0.34; 95% confidence interval [CI] = 0.18–0.64 (p = 0.028)). This would be thought to be useful for deciding the severity of the FNP patients in the outpatient clinic by evaluation of the early diagnosis and clinical course of FNP (**Table 3**).

However, in surgical FNP, the modified CS does not correspond. We could infer the course of the disease by modified CS, which increases in mild and chronic, but in the surgical group, modified CS decreases as time goes by and seems similar in disease severity with no significant difference.

As for lagophthalmos, there could be a compensatory reduction in mild and chronic, which was not significantly different, so it seems to be a projection of clinical course. However, regarding modified IPF, which increased, modified CS has not changed in the surgical group. Therefore, we can conclude that there would be less change in natural recovery in the surgical group (**Tables 4**, **5**).

The eyelid features of FNP patients of Korea were interestingly reported as lagophthalmos and ptosis rather than retraction which have been already known to be common in the western countries. Recovery of facial nerve function would be an important outcome for treatment recommendations. The initial severity of facial weakness provides valuable prognostic information for facial recovery. Patients with mild to moderate paresis have higher rates of recovery than those with severe or complete paresis (13). Most of the patients recovered without treatment, but considering the quality of life, conservative management is needed to reduce corneal exposure including eye drops and plugs. Using minimally invasive techniques, such as botulinum neurotoxin type A and dermal fillers, it would be way easier to treat the incomplete eyelid closure in patients in whom return of facial nerve function could be anticipated than the invasive ones such as tarsorrhaphy, gold plate insertion, nerve transfers, and eye sphincter substitution procedures.

A dermal filler can be used as a weight of the upper lid and elongate the lower lid. It has advantages of immediate application, being titratable, improved cosmesis, and reasonable duration of effects up to 9 months. For example, a 34-year-old woman was treated for FNP with upper- and lower-lid hyaluronic acid gel injection in the prelevator aponeurosis region and pretarsal region. After injection, modified CS, which reflects clinical features, improves and can predict improvement in the clinical course. When the filler could be loaded in the plane between the levator and Muller’s muscle in the upper eyelid and the septum in the lower eyelid, the modified CS was observed to recover close to the normal value after the injection (**Supplementary 1**).

The limitation of this study is the lack of combined lower eyelid in blink measurement in accordance with Ogawa’s study (14). Another limitation is that aberrant regeneration should be also considered such as hemifacial spasm, eye fissure narrowing during facial movement, and simple brow ptosis, even though we tried to exclude the patient in this study. Also, blinking is not the only factor to determine the severity of exposure keratopathy. Since this was a retrospective study with a relatively small number of patients, factors such as previous dry eye, lid laxity, and orbicularis tone should be considered. Further prospective studies with many more patients are needed.

In conclusion, despite these limitations, this study suggests meaningful indicators among blink profiles and dynamics, modified OS, and modified CS using modified IPF. Moreover, modified CS can be a useful tool for evaluating the early diagnosis and clinical course of FNP patients.

## Data availability statement

The raw data supporting the conclusions of this article will be made available by the authors, without undue reservation.

## Ethics statement

This study was reviewed and approved by CHAMC IRB 2018-12-016-002. Written informed consent for participation was not required for this study in accordance with the national legislation and the institutional requirements.

## Author contributions

Conceptualization and design of the study, YK, HL. Collection and management of data, YK. Analysis and interpretation of data, YK, HL. Writing of the article, YK, HL. Approval of the manuscript, YK, HL. All authors contributed to the article and approved the submitted version.

## Conflict of interest

The authors declare that the research was conducted in the absence of any commercial or financial relationships that could be construed as a potential conflict of interest.

## Publisher’s note

All claims expressed in this article are solely those of the authors and do not necessarily represent those of their affiliated organizations, or those of the publisher, the editors and the reviewers. Any product that may be evaluated in this article, or claim that may be made by its manufacturer, is not guaranteed or endorsed by the publisher.
